# Author Correction: Rewiring an olfactory circuit by altering cell-surface combinatorial code

**DOI:** 10.1038/s41586-025-10090-2

**Published:** 2026-01-04

**Authors:** Cheng Lyu, Zhuoran Li, Chuanyun Xu, Jordan Kalai, Liqun Luo

**Affiliations:** 1 Department of Biology and Howard Hughes Medical Institute, Stanford University, Stanford, CA USA; 2 Biology Graduate Program, Stanford University, Stanford, CA USA

**Keywords:** Axon and dendritic guidance, Neural circuits

Correction to: *Nature* 10.1038/s41586-025-09769-3 Published online 19 November 2025

In the version of the article initially published, in Fig. 1h, the bottom three panels in the “klg RNAi” column (second from right) were duplicates of the images in Fig. 1c. Fig. 1h has now been updated with the correct figures in the HTML and PDF versions of the article.

