# Supplementary material for: Rewiring an olfactory circuit by altering cell-surface combinatorial code
Source: Nature. 2025 Nov 19;649(8097):677–84. doi: 10.1038/s41586-025-09769-3 (PMC12804075; doi:10.1038/s41586-025-09769-3)
Supplement: Supplementary file 2 — Reporting Summary [file 41586_2025_9769_MOESM2_ESM.pdf]

Corresponding author(s): Liqun Luo

Last updated by author(s): Sep 17, 2025

# Reporting Summary

Nature Portfolio wishes to improve the reproducibility of the work that we publish. This form provides structure for consistency and transparency in reporting. For further information on Nature Portfolio policies, see our Editorial Policies and the Editorial Policy Checklist.

## Statistics

For all statistical analyses, confirm that the following items are present in the figure legend, table legend, main text, or Methods section.

| n/a | Confirmed | |
|---|---|---|
| ☐ | ☒ | The exact sample size (*n*) for each experimental group/condition, given as a discrete number and unit of measurement |
| ☐ | ☒ | A statement on whether measurements were taken from distinct samples or whether the same sample was measured repeatedly |
| ☐ | ☒ | The statistical test(s) used AND whether they are one- or two-sided<br>*Only common tests should be described solely by name; describe more complex techniques in the Methods section.* |
| ☐ | ☒ | A description of all covariates tested |
| ☐ | ☒ | A description of any assumptions or corrections, such as tests of normality and adjustment for multiple comparisons |
| ☐ | ☒ | A full description of the statistical parameters including central tendency (e.g. means) or other basic estimates (e.g. regression coefficient) AND variation (e.g. standard deviation) or associated estimates of uncertainty (e.g. confidence intervals) |
| ☐ | ☒ | For null hypothesis testing, the test statistic (e.g. *F*, *t*, *r*) with confidence intervals, effect sizes, degrees of freedom and *P* value noted<br>*Give P values as exact values whenever suitable.* |
| ☒ | ☐ | For Bayesian analysis, information on the choice of priors and Markov chain Monte Carlo settings |
| ☒ | ☐ | For hierarchical and complex designs, identification of the appropriate level for tests and full reporting of outcomes |
| ☒ | ☐ | Estimates of effect sizes (e.g. Cohen's *d*, Pearson's *r*), indicating how they were calculated |

*Our web collection on statistics for biologists contains articles on many of the points above.*

## Software and code

Policy information about availability of computer code

| | |
|---|---|
| Data collection | Two-photon imaging data were collected using PrairieView 5.4 (Bruker). Frame triggers and olfactory stimulus data were recorded as voltages on a Digidata 1550b (Molecular Devices) I/O board. Immunostained brains were imaged using a laser-scanning confocal microscope (Zeiss LSM 780). |
| Data analysis | Two photon imaging data were motion-corrected using non-rigid motion correction (NoRMCorre, v0.1.1) and then pre-processed using Fiji (to define regions of interest, version: 2.1.0/1.54j). All data were analyzed with custom code in python 2.7 and 3.6 and are available on github (https://github.com/Cheng-Lyu/CL_Stanford.git). |

For manuscripts utilizing custom algorithms or software that are central to the research but not yet described in published literature, software must be made available to editors and reviewers. We strongly encourage code deposition in a community repository (e.g. GitHub). See the Nature Portfolio guidelines for submitting code & software for further information.

## Data

Policy information about availability of data

All manuscripts must include a data availability statement. This statement should provide the following information, where applicable:

- Accession codes, unique identifiers, or web links for publicly available datasets
- A description of any restrictions on data availability
- For clinical datasets or third party data, please ensure that the statement adheres to our policy

All data are included in the manuscript and supplementary materials.

## Research involving human participants, their data, or biological material

Policy information about studies with human participants or human data. See also policy information about sex, gender (identity/presentation), and sexual orientation and race, ethnicity and racism.

| | |
|---|---|
| Reporting on sex and gender | Not applicable. |
| Reporting on race, ethnicity, or other socially relevant groupings | Not applicable. |
| Population characteristics | Not applicable. |
| Recruitment | Not applicable. |
| Ethics oversight | Not applicable. |

Note that full information on the approval of the study protocol must also be provided in the manuscript.

# Field-specific reporting

Please select the one below that is the best fit for your research. If you are not sure, read the appropriate sections before making your selection.

☒ Life sciences ☐ Behavioural & social sciences ☐ Ecological, evolutionary & environmental sciences

For a reference copy of the document with all sections, see nature.com/documents/nr-reporting-summary-flat.pdf

# Life sciences study design

All studies must disclose on these points even when the disclosure is negative.

| | |
|---|---|
| Sample size | No statistical tests were used to determine sample size. We used sample sizes (~6-20 flies per condition) that been previously shown to have sufficient statistical power in similar experiments in the past (e.g., Hong, Mosca, Luo 2012, Lyu, Abbott, Maimon 2022) |
| Data exclusions | We did not exclude flies or data from any analysis, unless brains stained for imaging appeared unsuitable (e.g., broken) at the time of imaging. |
| Replication | All experiments discussed in the paper were conducted on multiple animals with sample size specified. For most two-photon and behavioral experiments, data across multiple days were collected and the data across days were consistent. In immunohistochemistry plots, data across multiple days were collected and all imaged brains showed the same qualitative pattern of staining. |
| Randomization | Organisms are not allocated to control and experimental groups by the experimenter in this work, rather the flies' genotype determines their group. Thus, randomization of individuals into treatments groups is not relevant. |
| Blinding | The investigators were not blind to the flies' genotypes. All data collection and analysis were done computationally. During this process, data from control groups and experimental groups were analyzed equally using the same well-established protocols, therefore are less prone to investigator influence. |

# Reporting for specific materials, systems and methods

We require information from authors about some types of materials, experimental systems and methods used in many studies. Here, indicate whether each material, system or method listed is relevant to your study. If you are not sure if a list item applies to your research, read the appropriate section before selecting a response.

## Materials & experimental systems

| n/a | Involved in the study |
| --- | --- |
| ☐ | ☒ Antibodies |
| ☒ | ☐ Eukaryotic cell lines |
| ☒ | ☐ Palaeontology and archaeology |
| ☐ | ☒ Animals and other organisms |
| ☒ | ☐ Clinical data |
| ☒ | ☐ Dual use research of concern |
| ☒ | ☐ Plants |

## Methods

| n/a | Involved in the study |
| --- | --- |
| ☒ | ☐ ChIP-seq |
| ☒ | ☐ Flow cytometry |
| ☒ | ☐ MRI-based neuroimaging |

# Antibodies

| | |
| --- | --- |
| Antibodies used | rat anti-DNcad (from DSHB, RRID # AB_528121), chicken anti-GFP (from Aves Labs, RRID # AB_10000240), rabbit anti-DsRed (from Takara Bio, RRID # AB_10013483), and mouse anti-rat CD2 (1:200; OX-34, Bio-Rad) |
| Validation | All antibodies used in this study were validated as described at the following websites (and references therein): DSHB: https://dshb.biology.uiowa.edu/DN-Ex-8, Rockland: https://rockland-inc.com/store/Antibodies-to-GFP-and-Antibodies-to-RFP-600-901-215-O4L_23908.aspx, Takara: https://www.takarabio.com/products/antibodies-and-elisa/fluorescent-protein-antibodies/red-fluorescent-protein-antibodies?srsltid=AfmBOopUZqVextBqypoqsvRxsHH-H9rGlg0NFICn1UMie592NHF348BQ, Bio-Rad: https://www.bio-rad-antibodies.com/monoclonal/rat-cd2-antibody-ox-34-mca154.html?f=purified |

# Animals and other research organisms

Policy information about studies involving animals; ARRIVE guidelines recommended for reporting animal research, and Sex and Gender in Research

| | |
| --- | --- |
| Laboratory animals | We used male and female Drosophila melanogaster. All fly genotypes are described in details in the Methods. For behavioral experiments, female flies of the Canton-S strain were used, aged 3–5 days. In all other experiments, the w[1118] strain was used, with ages ranging from the pupal stage to 7 days old. |
| Wild animals | The study did not involve wild animals. |
| Reporting on sex | Most experiments were performed on both sexes and reached similar conclusion. Only the male-specific courtship behavioral experiments were performed using males. |
| Field-collected samples | The study did not involve samples collected from the field. |
| Ethics oversight | No ethical oversight was required because no vertebrates were used. |

Note that full information on the approval of the study protocol must also be provided in the manuscript.

# Plants

| | |
| --- | --- |
| Seed stocks | Not applicable. |
| Novel plant genotypes | Not applicable. |
| Authentication | Not applicable. |
